# Applicability of clinical genetic testing for deep brain stimulation treatment in monogenic Parkinson’s disease and monogenic dystonia: a multidisciplinary team perspective

**DOI:** 10.3389/fnins.2023.1282267

**Published:** 2023-11-10

**Authors:** Valentino Rački, Mario Hero, Eliša Papić, Gloria Rožmarić, Nada Starčević Čizmarević, Darko Chudy, Borut Peterlin, Vladimira Vuletić

**Affiliations:** ^1^Department of Neurology, Clinical Hospital Center Rijeka, Rijeka, Croatia; ^2^Department of Neurology, Faculty of Medicine, University of Rijeka, Rijeka, Croatia; ^3^Department of Medical Genomics and Biology, Faculty of Medicine, University of Rijeka, Rijeka, Croatia; ^4^Department of Neurosurgery, Clinical Hospital Dubrava, Zagreb, Croatia; ^5^Clinical Institute of Genomic Medicine, University Medical Centre Ljubljana, Ljubljana, Slovenia

**Keywords:** deep brain stimulation, dystonia, Parkinson’s disease, whole-exome sequencing, genetic testing

## Abstract

In this perspective article, we highlight the possible applicability of genetic testing in Parkinson’s disease and dystonia patients treated with deep brain stimulation (DBS). DBS, a neuromodulatory technique employing electrical stimulation, has historically targeted motor symptoms in advanced PD and dystonia, yet its precise mechanisms remain elusive. Genetic insights have emerged as potential determinants of DBS efficacy. Known PD genes such as *GBA*, *SNCA*, *LRRK2*, and *PRKN* are most studied, even though further studies are required to make firm conclusions. Variable outcomes depending on genotype is present in genetic dystonia, as DYT-TOR1A, NBIA/DYTPANK2, DYT-SCGE and X-linked dystonia-parkinsonism have demonstrated promising outcomes following GPi-DBS, while varying outcomes have been documented in DYT-THAP1. We present two clinical vignettes that illustrate the applicability of genetics in clinical practice, with one PD patient with compound *GBA* mutations and one *GNAL* dystonia patient. Integrating genetic testing into clinical practice is pivotal, particularly with advancements in next-generation sequencing. However, there is a clear need for further research, especially in rarer monogenic forms. Our perspective is that applying genetics in PD and dystonia is possible today, and despite challenges, it has the potential to refine patient selection and enhance treatment outcomes.

## Introduction

Deep brain stimulation (DBS) is a functional neuromodulatory procedure that entails the utilization of a neurostimulator to administer electrical impulses to the brain (Pycroft et al., 2018). For several decades, it has been utilized predominantly in the context of advanced Parkinson’s disease (PD) and dystonia to mitigate the motor symptoms associated with the conditions (Hickey and Stacy, 2016). The precise mechanisms underlying the therapeutic effectiveness of DBS have not been fully elucidated. Nevertheless, DBS has demonstrated benefits in a wide range of neurological and psychiatric disorders, including dystonia, essential tremor, Tourette syndrome, intractable pain, epilepsy, treatment-resistant depression, and obsessive-compulsive disorder (Ashkan et al., 2017). The technique is commonly employed to selectively focus on particular regions inside the brain, such as the subthalamic nucleus (STN), globus pallidus internus (GPi) or the thalamic ventral intermedial nucleus (VIM; Reese and Volkmann, 2017; Lozano et al., 2019). However, it is worth noting that the impact of DBS can be seen on whole neural networks, as it modulates activity in both upstream and downstream fashion (Ashkan et al., 2017). In this context, it has the potential to impact a wide range of symptoms, encompassing nonmotor symptoms observed in neurodegenerative disorders (Kurtis et al., 2017). There is a noted variability in patient outcomes with both mentioned conditions that is dependent on several factors. The main factors for outcomes are the target, accuracy of lead placement and stimulation parameters, which are more technical and can be mitigated by proper planning and procedure (Bari et al., 2015; Hartmann et al., 2019; Koeglsperger et al., 2019; Vitek et al., 2022). Other factors that influence outcomes are the disease subtypes and patient characteristics, specifically genetic background, which has increased in importance in recent years (Rizzone et al., 2019; Tisch, 2022).

Genetic basis of diseases cannot be discovered without testing, which is becoming an integral part of the clinical workup in neurological patients, especially in movement disorders or neuromuscular disease patients (Lefroy et al., 2020). Novel next generation sequencing techniques have made an enormous impact in genetic neurological disorders, and greatly expanded the diagnostic yields, while also leading to new gene discoveries in the field (Johnson, 2015). Still, even with these expanded capabilities, experts are still hesitant to routinely seek genetic testing for their patients with PD, with a recent study showing that 41 percent of movement disorder experts did not refer patients to genetic testing and more than 80 percent referred less than 11 patients in 1 year (Alcalay et al., 2020). Use of genetic testing in dystonia is more common than in PD, especially in younger patients, albeit still underutilized (Pozojevic et al., 2021). Cost–benefit of genetic testing is different depending on the medical field and specialty (Córdoba et al., 2018), and has not been established yet in movement disorders (Pal et al., 2023). However, it is important to note that the price for next generation sequencing has been decreasing with each new innovation in the field (Satam et al., 2023), and in some cases constituted a lesser cost compared to aggregated cost of various diagnostic procedures (Córdoba et al., 2018). Clinical algorithms have been put forth to guide clinical decisions on whether to test patients, but they have been focused on diagnostic yields, not therapeutic outcomes (Zech et al., 2021). Our team is comprised of neurologists, neurosurgeons and geneticists in three separate clinical centers. In this perspective, we offer our view of the applicability of genetic testing in dystonia and Parkinson’s disease patients planned to be, or treated with, deep brain stimulation, based on our experience combining the two fields in recent years, as well as two clinical vignettes that best exemplify our perspective.

## Parkinson’s disease

DBS has been used for advanced Parkinson’s disease treatment since the 1980’s (Frey et al., 2022), and it has a proven long-term benefit for patients properly selected for treatment (Limousin and Foltynie, 2019). The question of patient selection is an important one, and the current determinants include disease subtype, age, time to surgery, psychiatric comorbidities and cognitive issues (Rački et al., 2022). Genetic background of patients is another important determinant for disease progression, quality of life and outcome (Iwaki et al., 2019; Rizzone et al., 2019). Current evidence mainly encompasses the more common causative genes of PD, such as *GBA*, *SNCA*, *LRRK2*, and *PRKN*.

The most common genetic risk factor for PD are mutations in the *GBA* gene (Kovanda et al., 2022), and is the gene with the clearest evidence thus far, indicating that both STN and GPi DBS can have a solid impact on motor symptoms, albeit with an increased risk of cognitive decline than in noncarriers (Weiss et al., 2012; Lythe et al., 2017). This was confirmed in a recent composite analysis study with 366 subjects, where *GBA* mutation carriers that were treated with STN-DBS showed faster cognitive decline than *GBA* carriers with no DBS (Mattis Dementia rating Scale decline 1.71 points/year more), and even more so than noncarriers treated with DBS (Mattis Dementia rating Scale decline 1.49 points/year more; Pal et al., 2022). The patient groups were not evenly distributed but had enough participants (58 GBA + DBS+, 82 GBA + DBS-, 98 GBA-DBS+, 128 GBA-DBS-subjects), and the severity of the variant played a role in the speed of the decline. Even though there was a disparity in the number of patients in groups between the mild (non-neuronopathic) and severe (neuronopathic) variants with DBS (24 risk variant, 23 mild variant, 11 severe variant), it is important to keep in mind that there are differences between variants in clinical phenotype and therapeutic outcome (Pal et al., 2022). Regardless of risk, the benefit on motor outcomes and fluctuations is becoming clearer, as beneficial effects up to a 40% reduction have been seen on the Unified Parkinson’s Disease Rating Scale Part III (UPDRS III) and almost complete reduction (97%) in the Unified Parkinson’s Disease Rating Scale Part IV (UPDRS IV) scores (Angeli et al., 2013; Mangone et al., 2020). Finally, long-term follow-ups are important, as it has been shown that GBA-DBS patients can have axial deterioration and postural instability, despite positive earlier improvements (Straccia et al., 2022). Milder variants, like N370S, were shown to have beneficial effects with reduced incidence of cognitive or axial side effects compared to more severe variants like L444P (Artusi and Lopiano, 2023), highlighting that variant-to-variant differences will influence therapeutical decisions in the future. Furthermore, PD can be seen during the clinical presentation of other genetic diseases such as Gaucher’s disease type 1 (GD1), which is caused by homozygous or compound heterozygous *GBA* variants, with an age of onset after 60 years old and common cognitive impairment (Chetrit et al., 2013).

Various outcomes have been described for *SNCA* mutation carriers, with duplication patients initially displaying good to excellent motor outcomes (Antonini et al., 2012), but faster cognitive decline than noncarriers, especially in case of missense mutations (Youn et al., 2022). On the other hand, reports of *LRRK2* mutation carriers, especially G2019S, show that the response can be even better than in noncarriers (Sayad et al., 2016). Worse outcomes are found in R114G mutation carriers compared to other missense mutations in *LRRK2* (de Oliveira et al., 2019), indicating that we must consider both gene and mutation location when considering DBS in these patients. *PRKN* mutation carriers exhibit excellent long-term benefits of DBS in both STN and GPi, especially in patients with good responses to levodopa (Rizzone et al., 2019; Covolo et al., 2023), and can be considered good candidates for DBS. Patients that can show benefit from DBS are those with motor complications associated with levodopa treatment, with marked Unified Parkinsons’s Disease Rating Scale IV reduction (Aasly, 2020). For other, rarer mutations, such as *PINK1*, *DJ1*, *VPS35* and *CHCHD2*, the evidence is mostly based on case reports and is thus too early to give firm recommendations in routine practice. In general, we must keep in mind the benefits and potential side-effects of DBS therapy, such as postural instability (Ahrweiller et al., 2019) or selective cognitive decline (Rački et al., 2022), as these can be potentially exacerbated depending on certain monogenic causes like *DJ1*, *PRKN* or *SNCA* (Marsili et al., 2021). Taken together, we can see the value that genetic testing is beginning to have in PD, and we must keep in mind that patients with different variants in one gene can have different phenotypes and outcomes to advanced therapy. It is important to highlight that all evidence in this field is not based on randomized clinical trials, and further research is clearly needed, especially in rarer monogenic causes (Aasly, 2020; Kamo et al., 2022).

### Case vignette

The subject of this case vignette is a male patient, aged 43, who has been diagnosed with GD1, as a result of compound heterozygous mutations in the *GBA* gene (p.H294Q, p.N409S, p.D448H), which was previously published in Movement Disorders Clinical Practice (Racki et al., 2021). The patient had progressive parkinsonian symptoms characterized by marked tremor and rigidity, which responded to levodopa, but with early motor fluctuations and dyskinesias 3 years after initiating treatment. The utilization of DBS was suggested as a potential intervention to mitigate the progressive decline in the individual’s health status. Due to previous knowledge of possible limitations of treating *GBA* associated PD with DBS, we performed extensive neuropsychological testing. The results of cognitive examinations indicated the presence of mild depression, but no evidence of cognitive loss was observed. Consequently, based on these findings, the patient was deemed appropriate for STN-DBS. There was a notable and significant reduction in motor symptoms and dyskinesias after the procedure, resulting in a rapid favorable outcome. The overall levodopa dose was significantly reduced by 68%, with continuous improvements in the patient’s overall quality of life and a restoration of his independence. We decided to follow the cognitive functioning in this patient yearly through neuropsychological testing (Wechsler Memory Scale, Animal Naming Test, Controlled Oral World Association Test, various personality tests depending on age) and alternating different versions (8.1, 8.2, 8.3) of the Montreal Cognitive Assessment (MoCA), and testing throughout the last 5 years show an absence of any cognitive decline (Table 1). The improvements to quality of life and motor symptoms are still present as the patient is being able to care independently for his underage son. There are no issues with axial symptoms or other non-motor symptoms yet. This vignette highlights the potential advantages of initiating DBS at an early stage for individuals with GD1-PD who experience variable responses to levodopa. It emphasizes the importance of doing comprehensive cognitive evaluations prior to the treatment and regularly assessing cognitive functioning after DBS in patients with *GBA* mutations, and a way we can individualize treatment plans depending on the present genotype. Finally, to the best of our knowledge, this is the longest follow-up reported for a GD1-PD patient treated with DBS, as the previous reports mention great symptomatic improvement but no time course (Chetrit et al., 2013).

**Table 1:** Clinical characteristics of the GD1-PD patient through 5 years.

	MDS-UPDRS III	MDS-UPDRS IV	STN-DBS parameters		MoCA scores	LEDD
			Left	Right		
Before DBS/Med-ON	30	18	–	–	28	1,165
After DBS	10 (66.6%)	3 (83.4%)	8–1.5 V 90 μs 130 Hz	0–1.5 V 90 μs 130 Hz	27	375 (67.81%)
1st year	8 (73.4%)	1 (94.4%)	8–2.0 V 90 μs 130 Hz	0–1.9 V 90 μs 130 Hz	28	250 (78.54%)
2nd year	6 (80%)	1 (94.4%)	8–2.3 V 90 μs 130 Hz	0–2.9 V 90 μs 130 Hz	27	125 (89.27%)
3rd year	7 (23.3%)	1 (94.4%)	8–3.0 V 90 μs 130 Hz	0–2.5 V 90 μs 130 Hz	26	125 (89.27%)
4th year	9 (70%)	3 (83.4%)	8–3.5 V 90 μs 130 Hz	0–2.5 V 90 μs 130 Hz	26	125 (89.27%)
5th year	6 (80%)	1 (94.4%)	8–3.7 V 90 μs 130 Hz	0–2.5 V 90 μs 130 Hz	27	125 (89.27%)

All the MDS-UPDRS scores were calculated in Med-ON/Stim-ON conditions for each year of assessment. Percentage values reported in brackets refer to the percentage reduction of score from baseline (before DBS). DBS, deep brain stimulation; MDS-UPDRS, Movement Disorder Society Unified Parkinson’s Disease Rating Scale; STN, subthalamic nucleus; MoCA, Montreal Cognitivne Assessment; LEDD, levodopa equivalent daily dose; Med-ON, active phase of the medication; Stim-ON, turned on stimulations.

## Dystonia

Dystonia is defined as a movement disorder marked by sustained or intermittent muscle contractions that result in unusual, frequently repetitive movements, postures, or a combination of both (Albanese et al., 2013). Genetic forms of dystonia are defined as those wherein an underlying gene has been found as the source of the disease. As more and more dystonia-related genes are uncovered, our understanding of the genetic basis of the disorder has grown (Pozojevic et al., 2021). The most effective method for treating segmental and generalized dystonia that is refractory to medical treatment is globus pallidus internus deep brain stimulation (GPi DBS), which is suitable in children and adults (Tisch and Kumar, 2021). Shorter duration of the disease, younger age at onset and the specific subtype of dystonia are all predictors of the degree of improvement after GPi DBS surgery, where most cases of idiopathic isolated dystonia have better results than those with combined dystonia (Eltahawy et al., 2004; Andrews et al., 2010; Reese and Volkmann, 2014; Artusi et al., 2020). However, there are exceptions, such as acquired tardive dystonia caused on by exposure to neuroleptic drugs and dystonia noticed in the setting of neurodegeneration by brain iron accumulation (NBIA/DYT-PANK2; Tisch and Kumar, 2021). Also, idiopathic isolated craniofacial and laryngeal dystonia have a variable response to DBS, highlighting the importance the underlying cause and the body distribution when predicting the results of DBS for dystonia (Limotai et al., 2011; Finger et al., 2020; Tisch and Kumar, 2021). The response to DBS seems to differ among different monogenic forms of dystonia, showing varying degrees of clinical improvement for each. GPi-DBS has a positive effect on both short- and long-term motor and functional results in individuals with DYT-TOR1A, DYT-THAP1, and NBIA/DYTPANK2, in contrast to the other monogenic forms of dystonia, with DYT-TOR1A having the most significant clinical improvement (Artusi et al., 2020). Although DYT1 dystonia typically displays positive responsiveness to GPi-DBS, a study conducted by Cif et al. documented cases of secondary deterioration occurring after a few years among a subset of DYT1 dystonia patients who initially responded well at the 12-month mark (Cif et al., 2010). Similar was found in a study by Tsuboi et al., where results revealed that 11 out of 132 patients with DYT1 dystonia experienced secondary worsening within 6 months to 3 years following DBS. This decline was linked to an earlier age of onset, more rapid disease progression, and involvement of cranial regions (Tsuboi et al., 2020).

In contrast to Artusi et al., Tisch et al. described a worse and more variable effect of DBS in THAP1 dystonia (Tisch and Kumar, 2021). As per literature findings, the reported average improvement in Burke-Fahn-Marsden Dystonia Rating Scale (BFMDRS) is approximately 35%, and this can vary from 16 to 72% (Groen et al., 2010; Zittel et al., 2010; Panov et al., 2012; Krause et al., 2015). Additionally, there is a tendency for limited or negligible improvements in speech and bulbar function (Groen et al., 2010; Panov et al., 2012; Tisch and Kumar, 2021). Due to uncertainties regarding the efficacy of GPi DBS in THAP1 dystonia, alternative targets for deep brain stimulation are being investigated, such as the ventral lateral anterior thalamic nucleus (Mure et al., 2014). Several studies have shown that GPi DBS generally yields positive outcomes in the management of myoclonus dystonia arising from DYT-SCGE (DYT11) (Gruber et al., 2010; Kurtis et al., 2010; Kim et al., 2014). A study conducted by Kosutzka et al. presented 9 patients with genetically confirmed DYT-SCGE dystonia in whom GPi DBS achieved improvement in myoclonus by 94%, dystonia by 71 and 88% improvement in disability scores (Kosutzka et al., 2019). Favorable results due to GPi DBS have also been recorded in X-linked dystonia-parkinsonism (DYT3), with post-operative improvements in dystonia, alongside with improvements in the UPDRS III (Brüggemann et al., 2019). The effects of GPi DBS in other monogenic forms of both isolated and combined dystonia such as DYT-GNAL (DYT25), DYT-KMT2B (DYT28), DYT-ATP1A3 (DYT12), and DYT-ANO3 (DYT24) have been documented in smaller patient cohorts and showed different outcomes (Tisch and Kumar, 2021; Rajan et al., 2022). More specifically, patients with *GNAL* gene variants had faster responses than DYT6 patients, although the long term effects were similar and favorable in both reported groups (Ahn et al., 2019; Sarva et al., 2019). Even though GPi DBS is the most used and reported target, VIM DBS has also been reported as beneficial for dystonic tremor and myoclonus in DYT-SCGE, and can be used along with pallidal stimulation (Trottenberg et al., 2001; Wang et al., 2017). Due to the rare nature of genetic dystonia, most of the current findings are based on case reports and meta-analysis, which limit the interpretation of findings with a clear necessity for further investigations, like in PD.

### Case vignette

We present a 39-year-old woman with a novel likely pathogenic variant in the *GNAL* gene (NM_182978.4: c.394A > G: p.Lys132Glu). Pathogenicity of the variant was assessed using the ACMG/ACGS (American College of Medical Genetics and Genomics/Association for Clinical Genomic Science) criteria, based on the findings that the variant was not present in the control population, is located within the functional domain, has multiple *in-silico* predictors of pathogenicity and clinical picture that corresponds with *GNAL* patients (Fuchs et al., 2013). First symptoms began at the age of 7 years as the involuntary movements of the right hand and difficulties with fine motor skills, along with a mild cervical dystonia. Ten years later, during high school, the facial spasms started. The patient was initially treated at Clinical Hospital Center Zagreb, where she received botulinum toxin injections due to torticollis. The first control at our center in Rijeka occurred in 2016, when blepharospasm and cervical dystonia with writer’s cramp, action tremor and dystonic body posture were described. The patient was initially treated with 200 IU of botulinum toxin injections, but with consistent worsening. Due to the generalized dystonia and young age, DBS was proposed to the patient, which she refused. Several years after whole-exome sequencing was performed, and the results showed a heterogeneous variant in the *GNAL* gene. DBS was proposed due to the findings in the literature that this might be effective for her genotype (Ahn et al., 2019; Sarva et al., 2019; Tisch and Kumar, 2021). After through discussion with the patient, she agreed to the procedure and underwent bilateral directional GPi-DBS. She has a positive early response that improved even further in the first 6 months of treatment and now has a significant reduction in the BFMDRS from 40 preoperative (31 movement scale, 9 disability scale) to 10 (8 movement scale, 2 disability scale) 6 months after the procedure. Most beneficial were improvements in blepharospasm, cervical dystonia and posture. This vignette highlights the usefulness of genetic testing in routine clinical practice, which enabled the patient to make a better-informed decision, which was based on the currently available evidence.

## Conclusion

When initiating any diagnostic procedure, all physicians ask themselves what information can be gained and how it can be used to help our patients. The question of genetic testing is multifaceted and cannot be only approached through outcome, applicability or cost–benefit, as every patient has a basic right to the most accurate diagnosis if they choose to pursue it, which can in turn impact whole families. However, in this perspective, we wanted to highlight how genetics can be applied today to improve our decision making and treatment plans for PD and dystonia patients, and we believe it has a use in routine clinical practice.

DBS in PD has established long-term benefits for well-selected patients. Previously, patient selection involved factors like disease subtype, age, surgery timing, and cognitive issues, while recent studies suggest genetic influences on post-DBS outcomes. Genes like *GBA*, *SNCA*, *LRRK2*, and *PRKN* are the most common and studied. We can use the knowledge we have today to tailor our approaches to patients depending on their genotype, and it is part of our clinical practice. While various genetic factors impact DBS outcomes, evidence remains non-randomized, requiring further research, particularly for rarer genetic causes of the disease.

Genetic testing should be a standard in the assessment and management of dystonia patients due to the significant impact of underlying genetic mutations on treatment outcomes. This is especially apparent in cases where specific genetic forms of dystonia respond well to GPi DBS. According to the literature listed above, GPi DBS has shown varying degrees of success depending on the specific genetic subtype, some with significant clinical improvement. Genetic mutations of dystonia such as DYT-TOR1A, NBIA/DYTPANK2, DYT-SCGE and X-linked dystonia-parkinsonism have demonstrated promising outcomes following GPi-DBS, while varying outcomes have been documented in DYT-THAP1. For other genetic forms of dystonia, we still lack clinical data and studies with a larger number of subjects to determine the effectiveness of DBS therapy.

The choice of genetic test is still unclear in this rapidly changing field. Our pipeline generally involves whole-exome sequencing with multiplex-ligation dependent probe amplification to detect chromosomal abnormalities, which is a common approach (Olgiati et al., 2016). Even though we can use software defined panels in analysis for more accurate diagnostic procedures, performing whole-exome sequencing enables easier reassessment and adding novel genes as they are discovered. Whole-genome sequencing is currently used on a case-by-case basis depending on the complexity of phenotypes and the decision to find novel genes, which is what can be said for optical genome mapping as well. Targeted gene panels can be of use in larger scale testing, but defining and designing them can be challenging (Bean et al., 2020). While all procedures have a place in current testing pipelines, we must stress the importance of reassessment, and that patients should be informed that a current negative test can be changed in the future, especially in the case of variants of unknown significance (Salinas et al., 2020).

It must be said that the current evidence is still insufficient to make firm claims and many challenges remain, especially in cases of rarer monogenic causes, but we must be aware that the only way to improve is to continue to combine and try to apply new genetic findings for advanced treatment of movement disorders. This has to be highlighted in light of the findings that many specialists do not view this as applicable and consider it a purely scientific exercise, which we do not think is the case.

## Data availability statement

The original contributions presented in the study are included in the article/supplementary material, further inquiries can be directed to the corresponding author.

## Ethics statement

The studies involving humans were approved by Ethics Committee of the Clinical Hospital Center Rijeka. The studies were conducted in accordance with the local legislation and institutional requirements. The participants provided their written informed consent to participate in this study. Written informed consent was obtained from the individual(s) for the publication of any potentially identifiable images or data included in this article.

## Author contributions

VR: Conceptualization, Data curation, Investigation, Resources, Writing – original draft, Writing – review & editing. MH: Conceptualization, Data curation, Investigation, Resources, Writing – original draft, Writing – review & editing. EP: Data curation, Investigation, Methodology, Writing – review & editing. GR: Investigation, Methodology, Writing – original draft, Writing – review & editing. NČ: Investigation, Supervision, Writing – review & editing. DC: Investigation, Supervision, Writing – review & editing. BP: Conceptualization, Investigation, Supervision, Writing – review & editing. VV: Conceptualization, Funding acquisition, Investigation, Methodology, Resources, Supervision, Writing – review & editing.

## References

[ref1] Aasly J. O. (2020). Long-term outcomes of genetic Parkinson’s disease. J Mov Disord 13, 81–96. doi: 10.14802/jmd.19080, PMID: 32498494 PMC7280945

[ref2] Ahn J. H. Kim A. R. Kim N. K. D. Park W.-Y. Kim J. S. Kim M.. (2019). The effect of Globus pallidus Interna deep brain stimulation on a dystonia patient with the GNAL mutation compared to patients with DYT1 and DYT6. J Mov Disord 12, 120–124. doi: 10.14802/jmd.19006, PMID: 31158945 PMC6547035

[ref3] Ahrweiller K. Houvenaghel J. F. Riou A. Drapier S. Sauleau P. Haegelen C.. (2019). Postural instability and gait disorders after subthalamic nucleus deep brain stimulation in Parkinson’s disease: a PET study. J. Neurol. 266, 2764–2771. doi: 10.1007/s00415-019-09482-y, PMID: 31350641

[ref4] Albanese A. Bhatia K. Bressman S. B. DeLong M. R. Fahn S. Fung V. S. C.. (2013). Phenomenology and classification of dystonia: a consensus update: dystonia: phenomenology and classification. Mov. Disord. 28, 863–873. doi: 10.1002/mds.25475, PMID: 23649720 PMC3729880

[ref5] Alcalay R. N. Kehoe C. Shorr E. Battista R. Hall A. Simuni T.. (2020). Genetic testing for Parkinson disease: current practice, knowledge, and attitudes among US and Canadian movement disorders specialists. Genet. Med. 22, 574–580. doi: 10.1038/s41436-019-0684-x, PMID: 31680121 PMC7056638

[ref6] Andrews C. Aviles-Olmos I. Hariz M. Foltynie T. (2010). Which patients with dystonia benefit from deep brain stimulation? A metaregression of individual patient outcomes. J. Neurol. Neurosurg. Psychiatry 81, 1383–1389. doi: 10.1136/jnnp.2010.207993, PMID: 20841370

[ref7] Angeli A. Mencacci N. E. Duran R. Aviles-Olmos I. Kefalopoulou Z. Candelario J.. (2013). Genotype and phenotype in Parkinson’s disease: lessons in heterogeneity from deep brain stimulation. Mov. Disord. 28, 1370–1375. doi: 10.1002/mds.25535, PMID: 23818421 PMC3886301

[ref8] Antonini A. Pilleri M. Padoan A. Landi A. Ferla S. Biundo R.. (2012). Successful subthalamic stimulation in genetic Parkinson’s disease caused by duplication of the α-synuclein gene. J. Neurol. 259, 165–167. doi: 10.1007/s00415-011-6162-2, PMID: 21761143

[ref9] Artusi C. A. Dwivedi A. Romagnolo A. Bortolani S. Marsili L. Imbalzano G.. (2020). Differential response to pallidal deep brain stimulation among monogenic dystonias: systematic review and meta-analysis. J. Neurol. Neurosurg. Psychiatry 91, 426–433. doi: 10.1136/jnnp-2019-322169, PMID: 32079672

[ref10] Artusi C. A. Lopiano L. (2023). Should we offer deep brain stimulation to Parkinson’s disease patients with GBA mutations? Front. Neurol. 14:1158977. doi: 10.3389/fneur.2023.1158977, PMID: 37122287 PMC10140495

[ref11] Ashkan K. Rogers P. Bergman H. Ughratdar I. (2017). Insights into the mechanisms of deep brain stimulation. Nat. Rev. Neurol. 13, 548–554. doi: 10.1038/nrneurol.2017.105 28752857

[ref12] Bari A. A. Fasano A. Munhoz R. P. Lozano A. M. (2015). Improving outcomes of subthalamic nucleus deep brain stimulation in Parkinson’s disease. Expert. Rev. Neurother. 15, 1151–1160. doi: 10.1586/14737175.2015.1081815 26377740

[ref13] Bean L. Funke B. Carlston C. M. Gannon J. L. Kantarci S. Krock B. L.. (2020). Diagnostic gene sequencing panels: from design to report—a technical standard of the American College of Medical Genetics and Genomics (ACMG). Genet. Med. 22, 453–461. doi: 10.1038/s41436-019-0666-z, PMID: 31732716

[ref14] Brüggemann N. Domingo A. Rasche D. Moll C. K. E. Rosales R. L. Jamora R. D. G.. (2019). Association of Pallidal Neurostimulation and Outcome Predictors with X-linked dystonia parkinsonism. JAMA Neurol. 76:211. doi: 10.1001/jamaneurol.2018.3777 30508028 PMC6439955

[ref15] Chetrit E. B. Alcalay R. N. Steiner-Birmanns B. Altarescu G. Phillips M. Elstein D.. (2013). Phenotype in patients with Gaucher disease and Parkinson disease. Blood Cells Mol. Dis. 50, 218–221. doi: 10.1016/j.bcmd.2012.11.011 23265741

[ref16] Cif L. Vasques X. Gonzalez V. Ravel P. Biolsi B. Collod-Beroud G.. (2010). Long-term follow-up of DYT1 dystonia patients treated by deep brain stimulation: an open-label study: clinical course of DYT1 dystonia with DBS. Mov. Disord. 25, 289–299. doi: 10.1002/mds.22802, PMID: 20063427

[ref17] Córdoba M. Rodriguez-Quiroga S. A. Vega P. A. Salinas V. Perez-Maturo J. Amartino H.. (2018). Whole exome sequencing in neurogenetic odysseys: an effective, cost- and time-saving diagnostic approach. PLoS One 13:e0191228. doi: 10.1371/journal.pone.0191228, PMID: 29389947 PMC5794057

[ref18] Covolo A. Imbalzano G. Artusi C. A. Montanaro E. Ledda C. Bozzali M.. (2023). 15-year subthalamic deep brain stimulation outcome in a Parkinson’s disease patient with Parkin gene mutation: a case report. Neurol. Sci. 44, 2939–2942. doi: 10.1007/s10072-023-06789-7 37032388

[ref19] de Oliveira L. M. Barbosa E. R. Aquino C. C. Munhoz R. P. Fasano A. Cury R. G. (2019). Deep brain stimulation in patients with mutations in Parkinson’s disease-related genes: a systematic review. Mov Disord Clin Pract 6, 359–368. doi: 10.1002/mdc3.12795, PMID: 31286005 PMC6592794

[ref20] Eltahawy H. A. Saint-Cyr J. Giladi N. Lang A. E. Lozano A. M. (2004). Primary dystonia is more responsive than secondary dystonia to Pallidal interventions: outcome after Pallidotomy or Pallidal deep brain stimulation. Neurosurgery 54, 613–621. doi: 10.1227/01.NEU.0000108643.94730.21 15028135

[ref21] Finger M. E. Siddiqui M. S. Morris A. K. Ruckart K. W. Wright S. C. Haq I. U.. (2020). Auditory-perceptual evaluation of deep brain stimulation on voice and speech in patients with dystonia. J. Voice 34, 636–644. doi: 10.1016/j.jvoice.2019.02.010, PMID: 30879706 PMC6745002

[ref22] Frey J. Cagle J. Johnson K. A. Wong J. K. Hilliard J. D. Butson C. R.. (2022). Past, present, and future of deep brain stimulation: hardware, software, imaging, physiology and novel approaches. Front. Neurol. 13:825178. doi: 10.3389/fneur.2022.825178, PMID: 35356461 PMC8959612

[ref23] Fuchs T. Saunders-Pullman R. Masuho I. Luciano M. S. Raymond D. Factor S.. (2013). Mutations in GNAL cause primary torsion dystonia. Nat. Genet. 45, 88–92. doi: 10.1038/ng.2496, PMID: 23222958 PMC3530620

[ref24] Groen J. L. Ritz K. Contarino M. F. van de Warrenburg B. P. Aramideh M. Foncke E. M.. (2010). DYT6 dystonia: mutation screening, phenotype, and response to deep brain stimulation. Mov. Disord. 25, 2420–2427. doi: 10.1002/mds.23285, PMID: 20687191

[ref25] Gruber D. Kühn A. A. Schoenecker T. Kivi A. Trottenberg T. Hoffmann K.-T.. (2010). Pallidal and thalamic deep brain stimulation in myoclonus-dystonia. Mov. Disord. 25, 1733–1743. doi: 10.1002/mds.23312, PMID: 20623686

[ref26] Hartmann C. J. Fliegen S. Groiss S. J. Wojtecki L. Schnitzler A. (2019). An update on best practice of deep brain stimulation in Parkinson’s disease. Ther. Adv. Neurol. Disord. 12:1756286419838096. doi: 10.1177/1756286419838096, PMID: 30944587 PMC6440024

[ref27] Hickey P. Stacy M. (2016). Deep brain stimulation: a paradigm shifting approach to treat Parkinson’s disease. Front. Neurosci. 10:173. doi: 10.3389/fnins.2016.00173, PMID: 27199637 PMC4848307

[ref28] Iwaki H. Blauwendraat C. Leonard H. L. Liu G. Maple-Grødem J. Corvol J.-C.. (2019). Genetic risk of Parkinson disease and progression. Neurol Genet 5:e348. doi: 10.1212/NXG.0000000000000348, PMID: 31404238 PMC6659137

[ref29] Johnson N. E. (2015). Whole-exome sequencing in neurologic practice: reducing the diagnostic odyssey. Neurol. Genet. 1:e37. doi: 10.1212/NXG.0000000000000037, PMID: 27066574 PMC4811378

[ref30] Kamo H. Oyama G. Nishioka K. Funayama M. Hattori N. (2022). Deep brain stimulation for a patient with familial Parkinson’s disease harboring CHCHD2 p.T61I. Mov Disord Clin Pract 9, 407–409. doi: 10.1002/mdc3.13428, PMID: 35402650 PMC8974860

[ref31] Kim J. H. Na Y. C. Lee W. H. Chang W. S. Jung H. H. Chang J. W. (2014). Bilateral Globus pallidus Interna deep-brain stimulation in a patient with myoclonus-dystonia: a case report. Neuromodulation 17, 724–728. doi: 10.1111/ner.12162 24612290

[ref32] Koeglsperger T. Palleis C. Hell F. Mehrkens J. H. Bötzel K. (2019). Deep brain stimulation programming for movement disorders: current concepts and evidence-based strategies. Front. Neurol. 10:410. doi: 10.3389/fneur.2019.00410, PMID: 31231293 PMC6558426

[ref33] Kosutzka Z. Tisch S. Bonnet C. Ruiz M. Hainque E. Welter M.. (2019). Long-term GPi-DBS improves motor features in myoclonus-dystonia and enhances social adjustment. Mov. Disord. 34, 87–94. doi: 10.1002/mds.27474, PMID: 30302819

[ref34] Kovanda A. Rački V. Bergant G. Georgiev D. Flisar D. Papić E.. (2022). A multicenter study of genetic testing for Parkinson’s disease in the clinical setting. NPJ Parkinsons Dis. 8, 149–144. doi: 10.1038/s41531-022-00408-6, PMID: 36333361 PMC9636217

[ref35] Krause P. Brüggemann N. Völzmann S. Horn A. Kupsch A. Schneider G.-H.. (2015). Long-term effect on dystonia after pallidal deep brain stimulation (DBS) in three members of a family with a THAP1 mutation. J. Neurol. 262, 2739–2744. doi: 10.1007/s00415-015-7908-z 26486352

[ref36] Kurtis M. M. Rajah T. Delgado L. F. Dafsari H. S. (2017). The effect of deep brain stimulation on the non-motor symptoms of Parkinson’s disease: a critical review of the current evidence. NPJ Parkinsons Dis 3:16024. doi: 10.1038/npjparkd.2016.24, PMID: 28725706 PMC5516616

[ref37] Kurtis M. M. San Luciano M. Yu Q. Goodman R. R. Ford B. Raymond D.. (2010). Clinical and neurophysiological improvement of SGCE myoclonus–dystonia with GPi deep brain stimulation. Clin. Neurol. Neurosurg. 112, 149–152. doi: 10.1016/j.clineuro.2009.10.001 19896264 PMC2815107

[ref38] Lefroy H. Harrison V. Németh A. H. (2020). Genetic testing in neurology. Medicine 48, 545–549. doi: 10.1016/j.mpmed.2020.05.009

[ref39] Limotai N. Go C. Oyama G. Hwynn N. Zesiewicz T. Foote K.. (2011). Mixed results for GPi-DBS in the treatment of cranio-facial and cranio-cervical dystonia symptoms. J. Neurol. 258, 2069–2074. doi: 10.1007/s00415-011-6075-0, PMID: 21553081

[ref40] Limousin P. Foltynie T. (2019). Long-term outcomes of deep brain stimulation in Parkinson disease. Nat. Rev. Neurol. 15, 234–242. doi: 10.1038/s41582-019-0145-9 30778210

[ref41] Lozano A. M. Lipsman N. Bergman H. Brown P. Chabardes S. Chang J. W.. (2019). Deep brain stimulation: current challenges and future directions. Nat. Rev. Neurol. 15, 148–160. doi: 10.1038/s41582-018-0128-2, PMID: 30683913 PMC6397644

[ref42] Lythe V. Athauda D. Foley J. Mencacci N. E. Jahanshahi M. Cipolotti L.. (2017). GBA-associated Parkinson’s disease: progression in a deep brain stimulation cohort. J. Parkinsons Dis. 7, 635–644. doi: 10.3233/JPD-171172, PMID: 28777757

[ref43] Mangone G. Bekadar S. Cormier-Dequaire F. Tahiri K. Welaratne A. Czernecki V.. (2020). Early cognitive decline after bilateral subthalamic deep brain stimulation in Parkinson’s disease patients with GBA mutations. Parkinsonism Relat. Disord. 76, 56–62. doi: 10.1016/j.parkreldis.2020.04.002, PMID: 32866938

[ref44] Marsili L. Vizcarra J. A. Sturchio A. Dwivedi A. K. Keeling E. G. Patel D.. (2021). When does postural instability appear in monogenic parkinsonisms? An individual-patient meta-analysis. J. Neurol. 268, 3203–3211. doi: 10.1007/s00415-020-09892-3, PMID: 32436106

[ref45] Mure H. Morigaki R. Koizumi H. Okita S. Kawarai T. Miyamoto R.. (2014). Deep brain stimulation of the thalamic ventral lateral anterior nucleus for DYT6 dystonia. Stereotact. Funct. Neurosurg. 92, 393–396. doi: 10.1159/000365577 25359437

[ref46] Olgiati S. Quadri M. Bonifati V. (2016). Genetics of movement disorders in the next-generation sequencing era. Mov. Disord. 31, 458–470. doi: 10.1002/mds.26521, PMID: 26899883

[ref47] Pal G. Cook L. Schulze J. Verbrugge J. Alcalay R. N. Merello M.. (2023). Genetic testing in Parkinson’s disease. Mov. Disord. 38, 1384–1396. doi: 10.1002/mds.29500 37365908 PMC10946878

[ref48] Pal G. Mangone G. Hill E. J. Ouyang B. Liu Y. Lythe V.. (2022). Parkinson disease and subthalamic nucleus deep brain stimulation: cognitive effects in GBA mutation carriers. Ann. Neurol. 91, 424–435. doi: 10.1002/ana.26302, PMID: 34984729 PMC8857042

[ref49] Panov F. Tagliati M. Ozelius L. J. Fuchs T. Gologorsky Y. Cheung T.. (2012). Pallidal deep brain stimulation for DYT6 dystonia. J. Neurol. Neurosurg. Psychiatry 83, 182–187. doi: 10.1136/jnnp-2011-300979, PMID: 21949105

[ref50] Pozojevic J. Beetz C. Westenberger A. (2021). The importance of genetic testing for dystonia patients and translational research. J. Neural Transm. 128, 473–481. doi: 10.1007/s00702-021-02329-9, PMID: 33876307 PMC8099821

[ref51] Pycroft L. Stein J. Aziz T. (2018). Deep brain stimulation: an overview of history, methods, and future developments. Brain Neurosci Adv 2:2398212818816017. doi: 10.1177/2398212818816017, PMID: 32166163 PMC7058209

[ref52] Rački V. Hero M. Rožmarić G. Papić E. Raguž M. Chudy D.. (2022). Cognitive impact of deep brain stimulation in Parkinson’s disease patients: a systematic review. Front. Hum. Neurosci. 16:867055. doi: 10.3389/fnhum.2022.867055, PMID: 35634211 PMC9135964

[ref53] Racki V. Papic E. Almahariq F. Chudy D. Vuletic V. (2021). The successful three-year outcome of deep brain stimulation in Gaucher disease type 1 associated Parkinson’s disease: a case report. Mov Disord Clin Pract 8, 604–606. doi: 10.1002/mdc3.13185, PMID: 33981795 PMC8088117

[ref54] Rajan R. Garg K. Saini A. Radhakrishnan D. M. Carecchio M. BK B.. (2022). GPi-DBS for KMT2B-associated dystonia: systematic review and meta-analysis. Mov. Disord. Clin. Pract. 9, 31–37. doi: 10.1002/mdc3.13374, PMID: 35005062 PMC8721835

[ref55] Reese R. Volkmann J. (2014). “Deep brain stimulation for dystonia” in Deep Brain Stimulation: Technology and Applications, vol. 2 (Unitec House, 2 Albert Place, London N3 1QB, UK: Future Medicine Ltd), 18–31.

[ref56] Reese R. Volkmann J. (2017). Deep brain stimulation for the Dystonias: evidence, knowledge gaps, and practical considerations. Mov. Disord. Clin. Pract. 4, 486–494. doi: 10.1002/mdc3.12519, PMID: 30363085 PMC6090587

[ref57] Rizzone M. G. Martone T. Balestrino R. Lopiano L. (2019). Genetic background and outcome of deep brain stimulation in Parkinson’s disease. Parkinsonism Relat. Disord. 64, 8–19. doi: 10.1016/j.parkreldis.2018.08.006, PMID: 30121162

[ref58] Salinas V. Vega P. Marsili L. Pérez-Maturo J. Martínez N. Zavala L.. (2020). The odyssey of complex neurogenetic disorders: from undetermined to positive. Am. J. Med. Genet. C Semin. Med. Genet. 184, 876–884. doi: 10.1002/ajmg.c.31848, PMID: 33084218

[ref59] Sarva H. Trosch R. Kiss Z. H. T. Furtado S. Luciano M. S. Glickman A.. (2019). Deep brain stimulation in isolated dystonia with a GNAL mutation. Mov. Disord. 34, 301–303. doi: 10.1002/mds.27585 30536916 PMC6659994

[ref60] Satam H. Joshi K. Mangrolia U. Waghoo S. Zaidi G. Rawool S.. (2023). Next-generation sequencing technology: current trends and advancements. Biology (Basel) 12:997. doi: 10.3390/biology12070997, PMID: 37508427 PMC10376292

[ref61] Sayad M. Zouambia M. Chaouch M. Ferrat F. Nebbal M. Bendini M.. (2016). Greater improvement in LRRK2 G2019S patients undergoing subthalamic nucleus deep brain stimulation compared to non-mutation carriers. BMC Neurosci. 17:6. doi: 10.1186/s12868-016-0240-4, PMID: 26831335 PMC4736184

[ref62] Straccia G. Colucci F. Eleopra R. Cilia R. (2022). Precision medicine in Parkinson’s disease: from genetic risk signals to personalized therapy. Brain Sci. 12:1308. doi: 10.3390/brainsci12101308, PMID: 36291241 PMC9599944

[ref63] Tisch S. (2022). Deep brain stimulation in dystonia: factors contributing to variability in outcome in short and long term follow-up. Curr. Opin. Neurol. 35, 510–517. doi: 10.1097/WCO.0000000000001072 35787538

[ref64] Tisch S. Kumar K. R. (2021). Pallidal deep brain stimulation for monogenic dystonia: the effect of gene on outcome. Front. Neurol. 11:630391. doi: 10.3389/fneur.2020.630391, PMID: 33488508 PMC7820073

[ref65] Trottenberg T. Meissner W. Arnold G. Einhaupl K. M. Kupsch A. Kabus C.. (2001). Neurostimulation of the ventral intermediate thalamic nucleus in inherited myoclonus-dystonia syndrome. Mov. Disord. 16, 769–771. doi: 10.1002/mds.1119, PMID: 11481711

[ref66] Tsuboi T. Cif L. Coubes P. Ostrem J. L. Romero D. A. Miyagi Y.. (2020). Secondary worsening following DYT1 dystonia deep brain stimulation: a multi-country cohort. Front. Hum. Neurosci. 14:242. doi: 10.3389/fnhum.2020.00242, PMID: 32670041 PMC7330126

[ref67] Vitek J. L. Patriat R. Ingham L. Reich M. M. Volkmann J. Harel N. (2022). Lead location as a determinant of motor benefit in subthalamic nucleus deep brain stimulation for Parkinson’s disease. Front. Neurosci. 16:1010253. doi: 10.3389/fnins.2022.1010253, PMID: 36267235 PMC9577320

[ref68] Wang J.-W. Li J.-P. Wang Y.-P. Zhang X.-H. Zhang Y.-Q. (2017). Deep brain stimulation for myoclonus-dystonia syndrome with double mutations in DYT1 and DYT11. Sci. Rep. 7:41042. doi: 10.1038/srep41042, PMID: 28102337 PMC5244480

[ref69] Weiss D. Brockmann K. Srulijes K. Meisner C. Klotz R. Reinbold S.. (2012). Long-term follow-up of subthalamic nucleus stimulation in glucocerebrosidase-associated Parkinson’s disease. J. Neurol. 259, 1970–1972. doi: 10.1007/s00415-012-6469-7, PMID: 22427207

[ref70] Youn J. Oyama G. Hattori N. Shimo Y. Kuusimäki T. Kaasinen V.. (2022). Subthalamic deep brain stimulation in Parkinson’s disease with SNCA mutations: based on the follow-up to 10 years. Brain Behav. 12:e2503. doi: 10.1002/brb3.2503, PMID: 35040589 PMC8865141

[ref71] Zech M. Jech R. Boesch S. Škorvánek M. Necpál J. Švantnerová J.. (2021). Scoring algorithm-based genomic testing in dystonia: a prospective validation study. Mov. Disord. 36, 1959–1964. doi: 10.1002/mds.28614, PMID: 33949708

[ref72] Zittel S. Moll C. K. E. Brüggemann N. Tadic V. Hamel W. Kasten M.. (2010). Clinical neuroimaging and electrophysiological assessment of three DYT6 dystonia families. Mov. Disord. 25, 2405–2412. doi: 10.1002/mds.23279 20687193

